# Trajectory Planning for Data Collection of Energy-Constrained Heterogeneous UAVs

**DOI:** 10.3390/s19224884

**Published:** 2019-11-08

**Authors:** Zhen Qin, Chao Dong, Hai Wang, Aijing Li, Haipeng Dai, Weihao Sun, Zhengqin Xu

**Affiliations:** 1 College of Communications Engineering, Army Engineering University of PLA, Nanjing 210042, China; qzqzla912@163.com (Z.Q.);; 2 College of Electronic and Information Engineering, Nanjing University of Aeronautics and Astronautics, Nanjing 211106, China; 3 Department of Computer Science and Technology, Nanjing University, Nanjing 210023, China

**Keywords:** unmanned aerial vehicles, trajectory planning, sensors, data collection utility

## Abstract

Nowadays, Unmanned Aerial Vehicles (UAVs) have received growing popularity in the Internet-of-Things (IoT) which often deploys many sensors in a relatively wide region. Since the battery capacity is limited, sensors cannot transmit over a long distance. It is necessary for designing efficient sensor data collection mechanisms to prolong the lifetime of the IoT and enhance data collection efficiency. In this paper, we consider a UAV-enabled data collection scenario, where multiple heterogeneous UAVs with different energy constraints are employed to collect data from sensors. The value of data depends on the importance of the monitoring area of the sensor and the freshness of collected data. Our objective is to maximize the data collection utility by jointly optimizing the communication scheduling and trajectory of each UAV. The data collection utility is determined by the amount and value of the collected data. This problem is a variant of multiple knapsack problem, which is a classical NP-hard problem. First, we transform the initial problem into a submodular function maximization problem under energy constraints, and then we design a novel trajectory planning algorithm to maximize the data collection utility, while accounting for different values of data and different energy constraints of heterogeneous UAVs. Finally, under different network settings, the performance of the proposed trajectory planning algorithm is evaluated via extensive simulations. The results show that the proposed algorithm can obtain maximum data collection utility.

## 1. Introduction

Thanks to its tremendous application potentials in civilian, commercial and military related fields, the Internet of things (IoT) has attracted increased attention in many applications, e.g., natural disaster prediction, smart city, environmental monitoring, and reconnaissance [1,2,3,4,5]. The IoT often deploys many energy-constrained sensors in a relatively wide region. The task of the sensor is to collect data from the monitoring area, and then it uses multi-hop transmission mode to transmit data to the base station or sink node. Since the battery capacity is limited, sensors cannot transmit over a long distance. It is necessary for designing efficient sensor data collection mechanisms to prolong the lifetime of the IoT and enhance data collection efficiency [6].

In order to achieve efficient data collection, more and more people exploit Unmanned Aerial Vehicles (UAVs) to collect data from sensors, which will probably be a prospective technology for realizing the future IoT [6,7]. The heterogeneity and multi-domain nature of UAVs are indispensable in the IoT environment [8,9,10]. Thus, it is necessary for the IoT environment to use multiple heterogeneous UAVs with different capabilities. Different from traditional Wireless Sensor Networks (WSNs), the UAV-enabled data collection system uses mobile data collection devices installed on UAVs to communicate with sensors directly through the UAV-sensor channels, which are dominated by line-of-sight (LoS). UAVs can move towards the sensors and establish reliable connections with them due to their flexible deployment and high mobility [11]. UAV-enabled data collection system can reduce the energy consumption of sensors and improve the throughput and coverage.

There are many studies on studying how to use UAVs to collect reliable data from sensors. They mainly focus on two aspects of optimization. On the one hand, some works focus on solving the energy limitation problem of sensors in WSN [6,12,13,14]. They aim to optimize the wake-up schedule of sensor nodes, reduce the transmission power of sensors or improve the energy efficiency of data collection. However, these works rarely consider the value of data and distinguish the data collected from each sensor. For example, in reconnaissance application, the monitoring data of enemy command center is more important than that of living area. The value of data depends on the importance of monitoring area of the sensor and elapsed time after the previous collecting time (i.e., the freshness of collected data). On the other hand, some works focus on improving energy efficiency of UAV system [15,16,17]. They mainly aim to optimize the deployment of UAV, the trajectory of UAV, and the velocity of UAV. In these works, they use a single UAV or homogeneous UAVs. There are few studies that consider multiple heterogeneous UAVs with different energy constraints and power efficiency. Multiple heterogeneous UAVs can not only solve the energy limitation problem of a single UAV, but also fully utilize the capability characteristics of heterogeneous UAVs to implement complementary performance.

In this paper, we study a UAV-enabled data collection scenario, where multiple heterogeneous UAVs with different energy constraints are employed to collect data from sensors. The UAVs are responsible for transmitting data from sensors to the base station or sink node. We aim to maximize the data collection utility by planning trajectories of UAVs. The data collection utility is calculated by the value of data and amount of data. The value of data collected from the sensor depends on the importance of the monitoring area of the sensor and the freshness of collected data. Our problem contains three main technical challenges:

- Each UAV has different energy constraints and power efficiency. Thus, it is difficult to plan trajectory and assign tasks under their respective energy constraints.
- The value of data collected from each sensor is different, which depends on the importance of the monitoring area of the sensor and elapsed time after the previous collecting time (i.e., the freshness of collected data).
- Joint consideration of communication scheduling and trajectory optimization is a variant of the multiple knapsack problem, which is a classical NP-hard problem.

To solve this problem, we transform the initial problem into a submodular function maximization problem under energy constraints, and then, to maximize the data collection utility, we propose a novel trajectory planning algorithm. The main contributions of this paper are summarized as follows:

- Considering the different values of data and different energy constraints of heterogeneous UAVs, we focus on using multiple heterogeneous UAVs to collect data from sensors. Our objective is to maximize the data collection utility by jointly optimizing the communication scheduling and trajectories of UAVs. This problem is a variant of the multiple knapsack problem, which is a classical NP-hard problem [18,19].
- We prove that the data collection utility function is a submodular function, and transform the initial problem into the problem of maximizing a submodular function under energy constraints, and then we propose a novel trajectory planning algorithm to maximize the data collection utility, while accounting for different energy constraints of heterogeneous UAVs.
- Sufficient simulations are performed to demonstrate the validity and applicability of the proposed algorithm. The data collection utility of our algorithm can be increased by 134% at most, and the proposed algorithm is the closest to the optimal scheme compared with other algorithms.

The rest of this paper is organized as follows. In Section 2, we introduce the related work about the UAV-enabled data collection system and trajectory planning. In Section 3, we present the system model and problem formulation. Then we propose a solution for the formulated problem in Section 4. Simulation results are provided and analyzed in Section 5. Discussion is provided in Section 6. Finally, we conclude the paper in Section 7.

## 2. Related Work

### 2.1. UAV-Enabled Data Collection

There are many works on studying how to use the UAV to collect data from sensors. In [20], the authors considered that UAVs were used for collecting imagery information from nodes, and then, the UAVs transmitted information to the ground station. They proposed a predictive compression policy to maximize the end-to-end image quality. Gong et al. utilized a UAV to collect data from sensors which are deployed on a straight line [16]. The authors minimized the flight time of the UAV from a starting location to an ending location, and they jointly optimized the transmit power of sensors, the speed of the UAV and the data collection intervals. Ebrahimi et al. considered a scenario where UAVs collected the data in dense WSNs [6]. The authors used a novel solution methodology which is called projection-based Compressive Data Gathering (CDG). CDG aggregated data from sensors to the selected projection nodes which acted as cluster heads. Next, the UAV transferred the aggregated data from selected nodes to the sink node. In [21], the authors proposed a novel UAV-assisted backscatter communication. The UAV collected data from terrestrial backscattering tags, and then uploaded the collected data when it flied to the coverage area of the base station. Liu et al. proposed a UAV trajectory design for data collection to reduce redundant data and improving energy efficiency [22]. In [23], the authors deployed multi-UAV to serve vehicles on a highway. They utilized UAVs to deliver critical data to the vehicles crossing the given highway segment. By planning the trajectory of each UAV and optimizing the radio resource allocation, they aimed to minimize the number of UAVs to serve all vehicles. Sanaa et al. deployed UAVs as base stations to provide instant recovery via temporary wireless coverage [24]. They minimized the number of UAVs and optimized the positions of them in selected locations to enhance performance. Yang et al. studied a UAV-enabled data collection system, in which the UAV was employed to gather data from ground users. The sensors have limited battery and lower power. To prolong the lifetime of sensors, UAVs can move close to sensors to collect their information with minimum transmit power [11]. However, these works rarely consider distinguishing the data collected from each sensor. The value of data collected from each sensor is different, which depends on the importance of the monitoring area of the sensor and the freshness of collected data.

### 2.2. Trajectory Planning

Although people have strong interest in UAVs, studies on the location optimizing and trajectory planning of UAVs are still in progress. These studies are different in the optimization method and objective function because they assume different environments. These works are mainly divided into two types: single UAV trajectory planning and multi-UAV trajectory planning. Hu et al. considered a UAV used for the mobile edge computing system, where the mobile UAV equipped with computing resources provided service for many ground users [13]. By jointly optimizing the ratio of offloading tasks, the trajectories of UAVs, and the user scheduling variables, the authors minimized the maximum delay of all users. In the IoT system, Zhan et al. used a rotary-wing UAV for collecting the data from the IoT devices [14]. Under the energy constraint of the UAV, the authors minimized the maximum energy consumption of all IoT devices. Moataz et al. utilized a UAV to collect data from time-constrained IoT devices [25]. These devices with limited buffer sizes had their own target data upload deadline, and thus data needed to be collected before it lost its value. Their goal was to maximize the number of served IoT devices by jointly optimizing the radio resource allocation and the trajectory of the UAV. This paper took into account the change in the value of data. It provided a basis for us to consider the value of a sensor’s data. Hu et al. studied a UAV-enabled wireless power system, where the UAV provided wireless energy supply for ground users with a linear topology. The authors maximized the minimum received energy of ground users by optimizing the trajectory of the UAV [26]. They first presented the globally optimal one-dimensional (1D) trajectory solution to the minimum received energy maximization problem. Zeng et al. studied a multicasting system which utilized the UAV to transmit the file to all ground users [27]. By designing the UAV’s trajectory, the authors minimized the mission completion time of the UAV. Meanwhile, they guaranteed that each ground user can successfully recover the file. However, in some applications, a single UAV has been unable to meet the demands of missions. There are many works on studying how to design the trajectories of multi-UAV. Under urban environments, in order to minimize the risk to the population, the authors proposed a risk-aware trajectory planning algorithm for multi-UAV [28]. Islam et al. proposed a task-oriented trajectory planning scheme for multi-UAV [29]. The UAVs taken autonomous decisions to find their trajectories for flying to the mission area while avoiding collision to barriers. In [30], the authors aimed to minimizing the mission time by planning the trajectory of each UAV, while satisfying the time requirements. Under the same test scenarios, Christian et al. presented advancements over the A* and the smoothing algorithms [31]. Hu et al. exploited the nested Markov chains to analyze the probability for successful data transmission, and then, for real-time sensing missions, the authors proposed a sense-and-send protocol [32]. To solve the decentralized UAV trajectory planning problem, they proposed a multi-UAV Q-learning algorithm. Wu et al. used multi-UAV as mobile base stations which provided the service to the ground users [33]. The authors optimized the trajectory of each UAV to maximize the minimum throughput of ground users. Zhan et al. employed multi-UAVs to collect data from sensors in WSN [17]. By jointly optimizing the trajectories of UAVs, wake-up association and scheduling for sensors, the author minimized the maximum mission completion time of all UAVs. However, there are few studies that consider multiple heterogeneous UAVs with different energy constraints and power efficiency. Multi-heterogeneous UAVs not only can solve the energy limitation problem of a traditional single UAV, but also make use of the capability characteristics of heterogeneous UAVs to achieve complementary performance.

## 3. System Model and Problem Formulation

### 3.1. System Model

#### 3.1.1. Network Model

**Figure 1:**
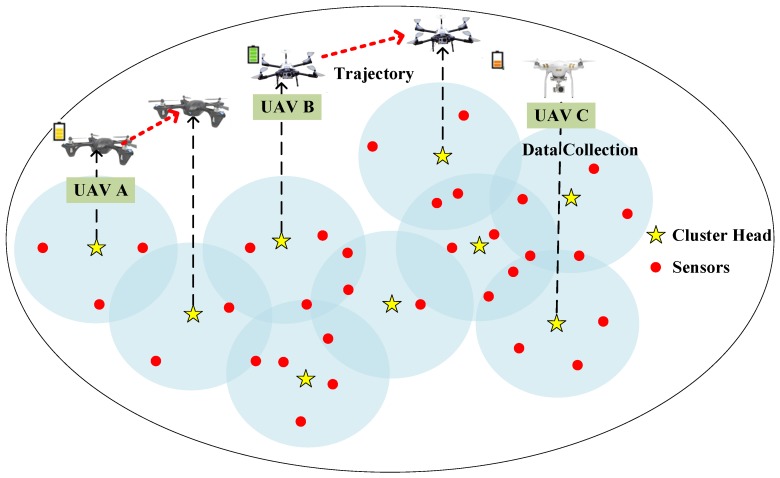
Monitoring scenario.

We consider a UAV-enabled data collection scenario, where *k* heterogeneous UAVs with different energy constraints are used for collecting the data from sensors to a remote base station or sink node as shown in Figure 1. In the UAV-enabled data collection system, since sensors are employed in a large area, it is inconvenient for the UAVs to fly over each sensor to collect data. In order to achieve efficient and scalable performance, more and more people adopt a clustering approach in WSN. In this paper, an overlapping clustering method is used for dividing the sensors on the ground [34,35]. Sensors transmit data to cluster heads, and then UAVs move towards cluster heads to collect data. The characteristic of overlapping clustering is that a sensor may belong to multiple clusters at the same time, which is different from traditional clustering algorithms. The cluster head can receive data from all sensors in its coverage. In other words, the sensor will transmit its data to each cluster head which it belongs to. For example, if a sensor fits in two overlapping clusters, it will transmit its data to two cluster heads. Establishing overlapping clusters can improve the success rate and robustness of data collection. For convenience, Table 1 provides major notations used in this paper.

The UAV, sensor and cluster head sets are denoted as $U=\{u_{1},..u_{i}..,u_{k}\}$, $S=\{s_{1},..s_{j}..,s_{n}\}$ and $C=\{c_{1},..c_{a}..,c_{m}\}$, respectively. In addition, ground sensors can be partitioned into *m* sets, $S_{1},S_{2},...S_{m}$. Each UAV $u_{i}$ is constrained with an energy budget $E_{\max,i}$. In this paper, the UAV mainly consumes communication-related energy and propulsion energy [36,37,38]. The communication-related energy is used for transmitting the collected data. The propulsion energy includes motion energy and hovering energy. The UAV consumes motion energy for flying between clusters, and hovering energy for hovering at cluster heads to collect data.

#### 3.1.2. Propulsion Energy Consumption Model

The motion energy is spent to overcome the gravity and drag forces caused by forward motions and wind. The motion energy consumption is calculated by minimum motion power $p_{\min,m}$ and the length of a UAV’s trajectory [38,39]. It can be expressed as

(1) $${E_{m}}_{,i}=\frac{p_{m,i}\cdot b_{i}}{v_{i}}=\frac{p_{\min,m}\cdot b_{i}}{\eta_{i}\cdot v_{i}},$$

where $p_{m,i}$ is the actual motion power consumption of UAV $u_{i}$, $\eta_{i}$ is the UAV’s power efficiency, $v_{i}$ is the velocity of the UAV $u_{i}$ and $b_{i}$ is the length of trajectory $L_{i}$.

The hovering energy consumption depends on the hovering time and actual hovering power $p_{h,i}$. The actual hovering power relates to the power efficiency and minimum hovering power $p_{\min,h}$. The minimum hovering power relates to the density of air, diameter, thrust and the number of rotors [39,40]. The hovering time is calculated by amount of data $N_{a,i}$ which is collected from cluster head $c_{a}$ by UAV $u_{i}$ and data transmission rate $R_{a,i}$ between cluster head $c_{a}$ and UAV $u_{i}$. Therefore, the hovering energy consumption of UAV $u_{i}$ for collecting data from cluster head $c_{a}$ can be calculated by

(2) $$E_{h,i}^{a}=\frac{p_{h,i}\cdot N_{a,i}}{R_{a,i}}=\frac{p_{\min,h}\cdot N_{a,i}}{\eta_{i}\cdot R_{a,i}}.$$

In this paper, we mainly consider that UAVs are used for data collection application. This kind of applications commonly used small rotary-wing UAVs. For example, the mass of UAV is 2.07 kg, the number of rotors is 4, and the rotor diameter is 0.254 m [38]. According to references [39,40], the minimum motion power is set to 388.32 J/s, and the minimum hovering power is set to 308 J/s.

#### 3.1.3. Communication-Related Energy Consumption Model

The communication-related energy consumption for transmitting data cannot be ignored when the transmission distance or the amount of data is large. The energy consumed for successful transmitting wireless data is affected by the channel between source and destination nodes, the transmission distance and other factors like interference, fading and noises. The communication energy consumption for transmitting $N_{a,i}$ bits over distance *d* can be calculated by [41]

(3) $$E_{c,i}^{a}=N_{a,i}\cdot d^{\alpha }\cdot e_{x},$$

where $e_{x}$ and α are constants which depends on the characteristics of the communication channel. $e_{x}$ is unit energy consumption which represents the energy consumption for transmitting one bit, measured in $J/(m^{\alpha }\cdot bit)$, and α is the path loss exponent which depends on the data transmission environment.

#### 3.1.4. Utility Model

The data collection utility is calculated by the value of data and the amount of data. The value of data depends on the importance of the monitoring area of the sensor and the freshness of collected data. In fact, the importance of the monitoring area has different performance metrics in different applications and scenarios. For example, the importance of the monitoring area can be defined by traffic [42].

To calculate the data collection utility, we first define the value of data. On the one hand, the value of data collected from the sensor depends on the importance of monitoring area of the sensor. In this paper, the initial value of data from $s_{j}$ is defined as $V_{j}^{0}=V_{j}^{\max}$. Once the data of sensor $s_{j}$ is collected, the value of data collected from $s_{j}$ is set to $V_{j}^{\min}$. For any sensor $s_{j}$ and $s_{j^{\prime }}$, if the monitoring area of sensor $s_{j}$ is more important than the monitoring area of sensor $s_{j^{\prime }}$, the relations can be expressed by

(4) $$V_{j}^{\max}>V_{j^{\prime }}^{\max},V_{j}^{\min}>V_{j^{\prime }}^{\min}.$$

On the other hand, the value of data collected from sensor $s_{j}$ depends on elapsed time after the previous collecting time (i.e., the freshness of collected data). For each sensor $s_{j}$, recovery interval $T_{j}$ is different which depends on the importance of the monitoring area and the required monitoring interval of the sensor. At time *t*, the value of data collected from sensor $s_{j}$ can be denoted as [43]

(5) $$V_{j}\left(t\right)=\left\{\begin{array}{c} A\times \exp\left(t-{t^{\prime }}_{j}\right)+B,\;if\;t-{t^{\prime }}_{j}\leq T_{j}\\ V_{j}^{\max},\;otherwise \end{array}\right.,$$

(6) $$\left\{\begin{array}{c} A=\frac{V_{j}^{\max}-V_{j}^{\min}}{e^{T_{j}}-1},\\ B=V_{j}^{\min}-A, \end{array}\right.$$

where ${t^{\prime }}_{j}$ is the time of previous data collection from sensor $s_{j}$. As we can see from Equation (5) and Equation (6), when the sensor’s data is collected by one UAV, the value of data will decrease to the minimum value. As time elapses, the value of data increases exponentially until it reaches its maximum value. After the value of data reaches the maximum value, it remains until the sensor’s data is collected by UAVs again.

In this paper, sensors transmit data to cluster heads, and then UAVs move towards cluster heads to collect data. The data collection utility mainly depends on the amount of data and its value. The data collection utility of the selected cluster head $c_{a}$ which is served by a UAV $u_{i}$ can be given by

(7) $$q_{a,i}=\sum_{s_{j}\in S_{a}}N_{a,i}\left(s_{j}\right)\cdot V_{j}\left(t_{a,i}\right),$$

where $S_{a}$ is the set of all sensors in cluster $c_{a}$, $N_{a,i}\left(s_{j}\right)$ represents the amount of data of sensor $s_{j}$ included in cluster head $c_{a}$ which is served by UAV $u_{i}$. Meanwhile, since the time of data collection is relatively short, we do not consider the changes of the data’s amount and value in the process of data collection. $t_{a,i}$ represents the time when a UAV $u_{i}$ starts to collect data from cluster head $c_{a}$. The data collection utility of UAV $u_{i}$ can be calculated by

(8) $$Q_{i}=\sum_{c_{a}\in P_{i}}q_{a,i},$$

where $P_{i}$ is the set of cluster head that is served by UAV $u_{i}$. Therefore, the overall utility of data collection mission can be calculated by

(9) $$Q\left(P\right)=\sum_{i=1}^{k}Q_{i}=\sum_{i=1}^{k}\sum_{c_{a}\in P_{i}}q_{a,i}.$$

### 3.2. Problem Formulation

In this paper, the flying altitude of the UAVs is assumed to be a constant altitude *H*. We assume that $r=(x_{r},y_{r},H)$ is the initial location of all UAVs. The total energy consumption $E_{i}$ includes the hovering energy consumption, motion energy consumption and communication energy consumption, which can be expressed by

(10) $$E_{i}=E_{m,i}+E_{h,i}+E_{c,i}.$$

Denote the trajectory of UAV $u_{i}$ projected on the ground as $l_{i}\left(t\right)={\left[x_{i}\left(t\right),y_{i}\left(t\right)\right]}^{T}\in$$R^{2\times 1}$, where 0≤t≤T. The trajectory of each UAV is subject to the velocity constraints, which can be given by

(11) $$∥\overset{\cdot }{l_{i}}\left(t\right)∥\leq v_{\max},\forall i,t\in \left[0,T\right],$$

where $\overset{\cdot }{l_{i}}\left(t\right)$ is the time derivative of $l_{i}\left(t\right)$ and $v_{\max}$ is the maximum velocity of UAVs.

Our goal is to plan the trajectories of heterogeneous UAVs with different energy constraints to maximize the overall data collection utility. Therefore, the optimization problem can be formulated as

(12) $$P1:\max_{P\in C,L_{i},1\leq i\leq k}\;Q\left(P\right)$$

(13) $$s.t.\;E_{i}\leq E_{\max,i},\forall i,$$

(14) $$\;\;\;\;\;\;\;∥\overset{\cdot }{l_{i}}\left(t\right)∥\leq v_{\max},\forall i,t\in \left[0,T\right],$$

(15) $$\;\;\;\;\;L_{i}\left(0\right)=L_{i}\left(T\right)=r,\forall i,$$

(16) $$\;\;\;\;\;\;\;\;\;\;\;\;\;∥L_{i}\left(t\right)-L_{i^{\prime }}\left(t\right)∥\geq d_{\min},\forall i\neq i^{\prime },t\in \left[0,T\right].$$

where *P* represents the selected cluster heads, $L_{i}$ represents the trajectory of UAV $u_{i}$, *r* is the initial location of all UAVs and $d_{\min}$ denotes the minimum distance between UAVs to ensure collision avoidance. Constraint (13) implies the energy consumption of UAV $u_{i}$ cannot be greater than its maximum energy constraint $E_{\max,i}$. In (15), it ensures that each UAV needs to return to initial location *r* by the end of data collection mission. When all UAVs fly at the same altitude *H*, the trajectories of UAVs are also constrained by collision avoidance (16).

## 4. Solution

### 4.1. Hardness Analysis

The formulated problem combines two-level optimizations. The objective of upper level optimization is to select cluster heads and the objective of lower level optimization is to design trajectories for energy-constraint heterogeneous UAVs. The results of each level optimization problem would directly affect another level optimization. If we select cluster heads without considering trajectory planning, it will consume much motion energy. If we do not consider to select appropriate cluster heads in trajectory planning, the data collection utility will not be maximized. Therefore, the two-level optimizations are coupled with each other and cannot be solved separately.

Without considering the motion energy consumption, the upper cluster head selection problem can be regarded as a simplified form of the formulated problem

(17) $$P2:\max_{P\in C,L_{i},1\leq i\leq k}\;Q\left(P\right)$$

(18) $$s.t.\;E_{h,i}+E_{c,i}\leq E_{\max,i},\forall i,$$

(19) $$\;\;\;\;\;∥\overset{\cdot }{l_{i}}\left(t\right)∥\leq v_{\max},\forall i,t\in \left[0,T\right],$$

(20) $$\;\;\;L_{i}\left(0\right)=L_{i}\left(T\right)=r,\forall i,$$

(21) $$\;\;\;\;\;\;\;\;\;\;\;∥L_{i}\left(t\right)-L_{i^{\prime }}\left(t\right)∥\geq d_{\min},\forall i\neq i^{\prime },t\in \left[0,T\right].$$

This problem can be modeled as a multiple capacity-constraint knapsack problem. When k=1, this problem is a knapsack problem, which is a classical NP-hard problem. Therefore, when the value of *k* is greater than 1, our problem is also NP-hard. The knapsack problem is a combinatorial optimization problem: under the given weight limit, its objective is to select items which have unique weight and value to maximize the total value [18,19].

In addition, if we consider the motion energy consumption, we would calculate the *k* closed trajectories including all cluster heads in the selected set *P*. This problem can be formulated as multiple Travelling Salesman Problem, which is also a NP-hard problem. Therefore, the original optimization problem is difficult to solve, which combines two coupling NP-hard problems.

### 4.2. Submodular Analysis

To solve this problem, we transform the initial problem into the problem of maximizing a submodular function with energy constraints. We prove the data collection utility function has three tractable properties: submodularity, nonnegativity and monotonicity. We first give some definitions to facilitate further analysis.

**Definition** **1.** *(monotonicity, nonnegativity, and submodularity) given a finite set Ψ, a submodular function is a set function $f:2^{\Psi }\to R$. *f* is called monotonicity (nondecreasing), nonnegativity, and submodularity if and only if it can satisfy the following requirements, respectively.*

- *f(X)≤f(Y) for all X⊆Y⊆Ψ or equivalently: f(X∪{x})−f(X)>0 for all X⊆Ψ and x∈Ψ\X (monotonicity);*
- *f(∅)=0 and f(X)≥0 for all X⊆Ψ (nonnegativity);*
- *f(X)+f(Y)≥f(X∪Y)+f(X∩Y), for any X,Y⊆Ψ or equivalently: f(X∪{x})−f(X)≥f(Y∪{x})−f(Y), X⊆Y⊆Ψ,x∈Ψ\Y (submodularity).*

**Theorem** **1.** *The constructed objective function is submodular, monotone and nonnegative.*

**Proof.**  According to the definition of the data collection utility function, Q(P)≥0, then it is nonnegative.

The data collection utility Q(P) increases as the number of cluster heads collected by UAVs increases. According to the utility model, for the set X⊆Y⊆C, we can obtain the following inequation

(22) Q(X)≤Q(Y),

where implies Q(P) is monotone. Next, we prove that Q(P) is a submodular function by proving the following inequation

(23) Q(X∪{c})−Q(X)≥Q(Y∪{c})−Q(Y),X⊆Y⊆C,x∈C\Y,

where *X* and *Y* represent the set of cluster heads in WSN. We denote $S_{X}$ as the sensors covered by the set of cluster heads *X*. We prove the inequation under two cases.

*Case 1* ($S_{c}\cap S_{Y}=\emptyset$): In this case, the data of sensors included in the newly added cluster has never been collected. Therefore, the value of data of sensors covered by cluster head *c* can reach their maximum value $V^{\max}$. We can obtain

(24) Q(X∪{c})−Q(X)=Q(Y∪{c})−Q(Y).

*Case 2* ($S_{c}\cap S_{Y}\neq \emptyset$): In this case, the data of some sensors included in the newly added cluster has been collected. Once the data of sensor is collected, the value of data will be set to $V^{\min}$. Therefore, we can obtain

(25) Q(X∪{c})−Q(X)≥Q(Y∪{c})−Q(Y).

Therefore, we prove that Q(P) is a submodular function. To solve this problem, the initial problem is transformed into a submodular function maximization problem with energy constraints. We propose a novel trajectory planning algorithm which refers to the idea of [44,45]. It aims to maximize the overall data collection utility, while accounting for cluster head selection and differnent energy constraints of heterogeneous UAVs. □

### 4.3. Algorithm

Based on the submodular function, we jointly consider the upper level optimization and lower level optimization, and then we design a simple but efficient algorithm referring to the idea of [44,45]. The algorithm attempts to select appropriate cluster heads to collect data and design the collecting sequence. The core idea of our algorithm is to iteratively select a new cluster head $c_{j^{\prime }}$ by greedy method, which has the maximum utility-cost ratio. For example, in iteration *j*, the selected cluster head can be expressed as follows

(26) $$c_{j^{\prime }}=\underset{c\in I\backslash P_{j-1}}{\arg\max}\frac{Q\left(P_{j-1}\cup \left\{c\right\}\right)-Q\left(P_{j-1}\right)}{E\left(P_{j-1}\cup \left\{c\right\}\right)-E\left(P_{j-1}\right)}.$$

Algorithm 1 consists of a parent loop and a child loop. After inputting and initializing relative parameters, we use the parent loop to select cluster heads and plan trajectories (Line 2–Line 12). When all cluster heads have been traversed, the parent loop is no longer executed. In the parent loop, there is a child loop for selecting the cluster head which has the maximum utility-cost ratio (Line 3–Line 7). In each iteration, we use Algorithm 2 to calculate the energy cost and the trajectory, which considers the energy constraint of each UAV. The utility and energy cost are calculated according to the previously selected cluster heads plus possible cluster head $c_{j}$, and then, we pick up the cluster head with the highest utility ratio (Line 7). Afterwards, we check whether the UAVs satisfy the respective energy constraints (Line 8–Line 10). Next, it deletes this cluster head and starts next parent loop. Each parent loop returns a solution which is better than previous solution and the nature of the result depends on the quality of Algorithm 2. Finally, we can obtain the selected cluster heads *P* and the trajectories of UAVs *L* which satisfy the respective energy constraints.

**Algorithm 1** Cluster Head Selection and Trajectory Planning Algorithm
**Input:** Cluster head set *C*, energy constraints $E_{\max,i},1\leq i\leq k$.**Output:** Selected cluster heads P⊆C and *k* trajectories of UAVs.1:Initialize I←C, $P_{0}\leftarrow \emptyset$, $E(P_{0})\leftarrow 0$, $Q(P_{0})\leftarrow 0$, L←∅, j←1;2:
**while** I≠∅ **do**
3: **for** i=1 to $\left|I\right|$ **do**4:  Calculate $Q(P_{j-1}\cup \left\{c_{i}\right\})$ and $Q(P_{j-1})$;5:  Using Algorithm 2 to get the trajectories and the energy cost $E(P_{j-1}\cup \left\{c_{i}\right\})$;6: **end for**7: $c_{j^{\prime }}=\underset{c\in I}{\arg\max}\frac{Q\left(P_{j-1}\cup \left\{c\right\}\right)-Q\left(P_{j-1}\right)}{E\left(P_{j-1}\cup \left\{c\right\}\right)-E\left(P_{j-1}\right)}$;8: **if** $E(P_{j-1}\cup \left\{c_{j^{\prime }}\right\})\neq Inf$ **then**9:  $P_{j}\leftarrow P_{j-1}\cup \left\{c_{j^{\prime }}\right\},j\leftarrow j+1,L\leftarrow L_{c_{j^{\prime }}}$;10: **end if**11: $I\leftarrow I\backslash c_{j^{\prime }}$;12:
**end while**
13:Output $P\leftarrow P_{j-1}$, *L*.


**Algorithm 2** Multiple Energy-constrained Heterogeneous UAV Trajectory Planning Algorithm
**Input:** *P*, $E_{\max,i},1\leq i\leq k$, starting location *r*.**Output:** *k* trajectories of UAVs and the energy cost *E*.1:Initialize Y←P, E←∅, MAXE=0, L←∅;2:Sort the UAVs, based on $E_{\max,i}$, in descending order in a list ζ;3:**while**Y≠∅ && MAXE≠Inf **do**4: **for** i=1 to $\left|\zeta \right|$ **do**5:  Initialize $X_{i}\leftarrow \left\{r\right\},L_{i}\leftarrow \emptyset ,E_{i}\leftarrow 0$;6:  **for** j=1 to $\left|Y\right|$ **do**7:   Use the TSP algorithm to calculate the energy cost $E_{i,j}$ and the trajectory $L_{i,j}$ which covers all cluster heads in $X_{i}\cup c_{j}$;8:   **if** $E_{i.j}\leq E_{\max,i}$ **then**9:    $X_{i}\leftarrow X_{i}\cup c_{j},E_{i}\leftarrow E_{i,j},L_{i}\leftarrow L_{i,j}$;10:   **end if**11:  **end for**12:  $Y\leftarrow Y\backslash X_{i},E\leftarrow E+E_{i},L\leftarrow L\cup L_{i}$;13: **end for**14: **if** Y≠∅ **then**15:  E=Inf;16: **end if**17:
**end while**



## 5. Simulation Results

In this paper, our algorithm aims to maximize the data collection utility by optimizing the trajectory of each UAV. The data collection utility is calculated by the value and amount of data. The value of data depends on the importance of the monitoring area of the sensor and the freshness of collected data.

### 5.1. Simulation Setup

We consider a mission area of size 1 km × 1 km. The simulations are performed according to parameters specified in Table 2 [38,39,40,41]. The time requirement for data uploading is not a constant. It can be changed depending on the amount of data and data transmission rate. In fact, sensors continuously monitor the area and generate new data. However, since the time of data collection is relatively short, we do not consider the changes of data’s amount and value in the process of data collection. We assume that communication links between UAVs and sensors are dominated by the LoS links where the channel quality mainly depends on the UAV-sensor distance [16,33,46]. Meanwhile, since the UAVs fly at a fixed altitude, we can set the data transmission rate to be 2 Mbps. Furthermore, the simulation results are averaged over extensive simulation runs.

### 5.2. Baseline Setup

To demonstrate the performance of the proposed UAV trajectory planning algorithm (UE), we compare and implement the following four benchmark schemes:

- Optimal scheme (OPT): To evaluate how the proposed algorithm approaches the optimal performance, we use brute-force searching method to get the optimal scheme which maximizes the data collection utility.
- RAN algorithm: Multi-UAV randomly select cluster heads for data collection. Based on the selected data collection points, we consider the energy constraint of each UAV to plan the trajectories.
- EC algorithm: The main purpose of this algorithm is to collect as much data as possible from sensors. However, it does not take into account the value of data.
- GU algorithm: This algorithm selects a cluster head which has maximum data collection utility in each iteration. However, the algorithm does not consider the energy consumption of the UAV when it selects cluster heads.

### 5.3. Different Number of Sensors

**Figure 2:**
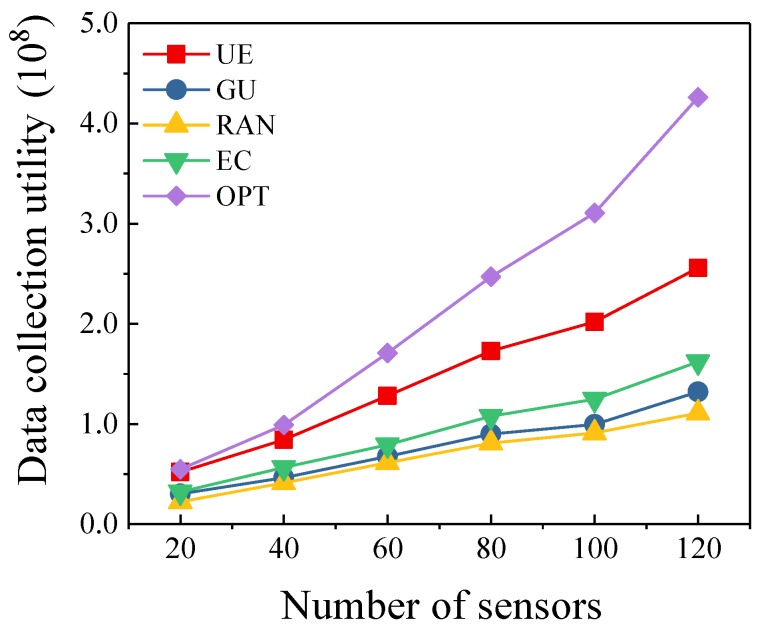
Different Number of Sensors.

In this simulation, we set the number of UAVs to 3. Figure 2 shows the trend of data collection utility as the number of sensors changes. We can observe that the data collection utility gradually increases as the number of sensors increases. Our algorithm achieves almost the same performance with the optimal scheme when the number of sensors is small. However, the gap between our algorithm and the optimal scheme increases as the number of sensors increases, from 5.2% to 66.6%. Meanwhile, the proposed algorithm shows better performance when the number of sensors is large. Compared with RAN algorithm, the data collection utility of our algorithm is improved by 103%–134%. This is reasonable since it chooses the data collection points with the highest utility-cost ratio each time, which saves the energy of UAVs and improves the data collection utility of UAVs. Compared with EC algorithm, the data collection utility of our algorithm is improved by 49%–62%. Because our algorithm considers the value of data when selecting cluster heads, not only the amount of data collection. Furthermore, GU algorithm chooses a cluster which has the most data collection utility. However, it does not consider the energy consumption for collecting data from this cluster. Compared with the GU algorithm, the data collection utility of our algorithm is improved by 72%–102%. Our proposed algorithm makes reasonable and effective use of UAV’s energy to collect more valuable data of sensors.

**Figure 3:**
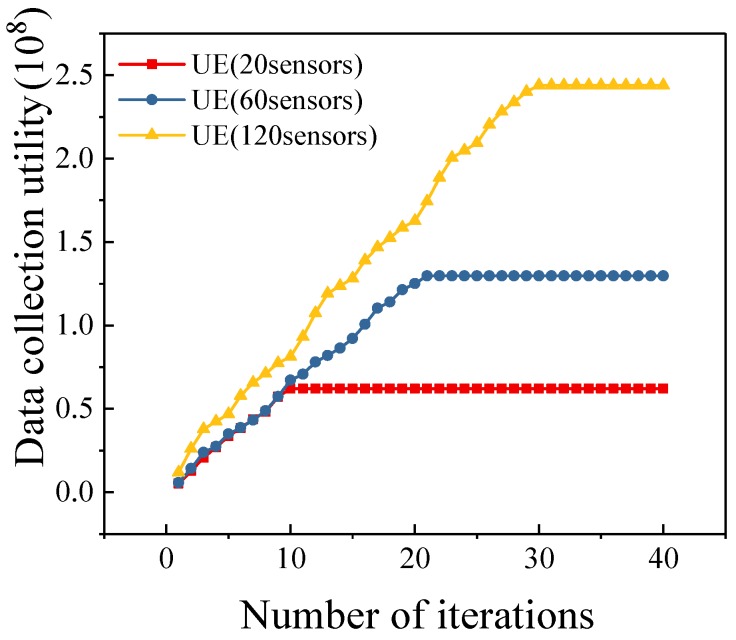
Convergence of UE Algorithm (n=20,60,120.)

In Figure 3, we illustrate the convergence of our algorithm under different number of sensors. From the figure, we note that our algorithm achieves fast convergence in three cases. Meanwhile, we can obtain that the number of iterations of the proposed algorithm is related to the number of sensors.

### 5.4. Different Number of UAVs

**Figure 4:**
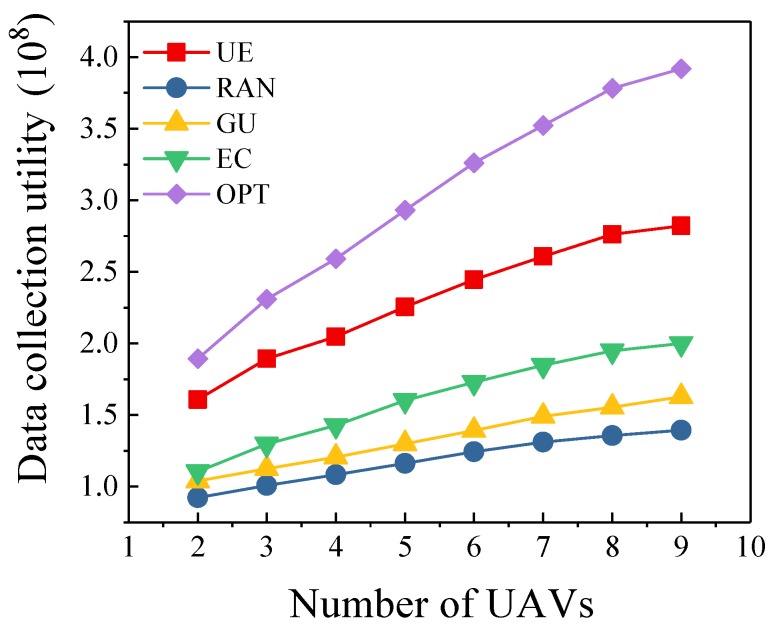
Different Numberof UAVs.

**Figure 5:**
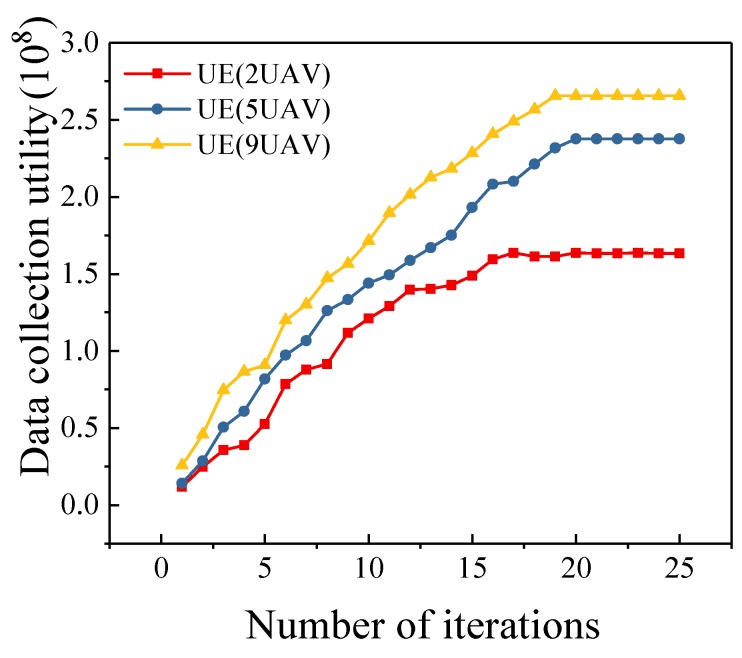
Convergence of UEAlgorithm (k=2,5,9).

In this simulation, the number of sensors is set to 100. As shown in Figure 4, as the number of UAVs increases, the advantages of our algorithm are more obvious. This is because we fully consider the energy constraint and power efficiency of each UAV in trajectory planning. Therefore, as the number of heterogeneous UAVs increases, the data collection utility gradually increases, and our algorithm is closer to the optimal scheme than other three algorithms. The data collection utility of the optimal scheme is 17.6%–38.9% higher than that of our algorithm. In practical applications, we should consider the mission requirements and existing equipment to dispatch an appropriate number of UAVs to perform mission. Using too many UAVs may bring economic pressure and reduce the energy efficiency. Figure 5 shows the convergence of the proposed algorithm under different number of UAVs. We can observe that our algorithm achieves fast convergence. Meanwhile, we can also find that the number of UAVs has little effect on the convergence of the proposed algorithm.

### 5.5. Different Mission Area Sizes

**Figure 6:**
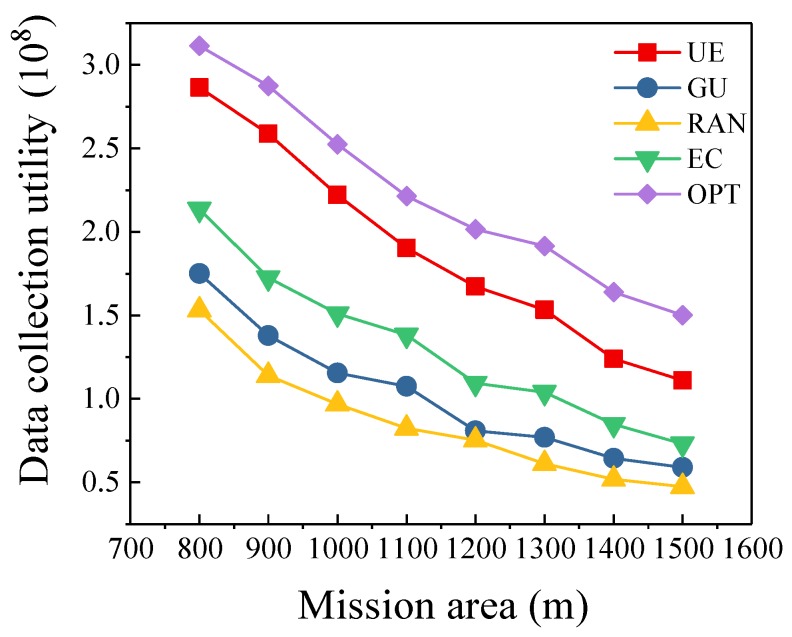
Different Mission Area Sizes.

**Figure 7:**
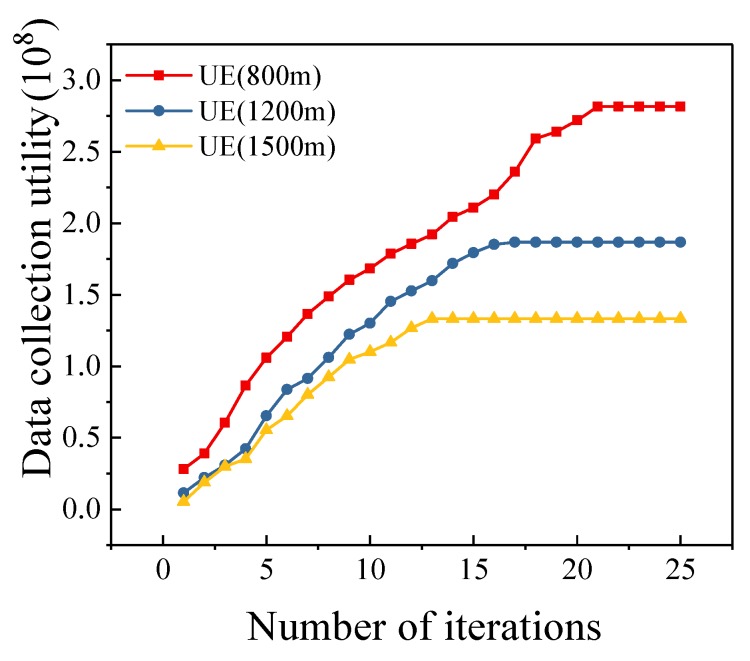
Convergence of UE Algorithm (800 m, 1200 m, 1500 m).

Figure 6 shows the trend of data collection utility as the size of mission area changes. We set the number of sensors to 100, and the number of UAVs to 3. We assume the mission area is a square area, and the variable in this simulation is the side length of the mission area. As shown in Figure 6, with the expansion of the mission area, the data collection utility gradually decreases. This is reasonable since the UAVs need to consume more energy to fly between data collection points when the sensors are distributed in a large mission area. Under this scenario, the energy used for data collection will be reduced, leading to the decrease of data collection utility. However, when the mission area is large, the data collection utility of our algorithm is also higher than other algorithms. For example, when the mission area is 1500 m × 1500 m, the data collection utility of our algorithm is 52%–134% higher than compared algorithms. Meanwhile, the data collection utility of the optimal scheme is 8.7%–35.1% higher than that of our algorithm as the mission area expands. In Figure 7, we illustrate the convergence of our algorithm under different mission area sizes. We can see that the algorithm can converge quickly in different data collection areas.

### 5.6. Trajectories of UAVs

**Figure 8:**
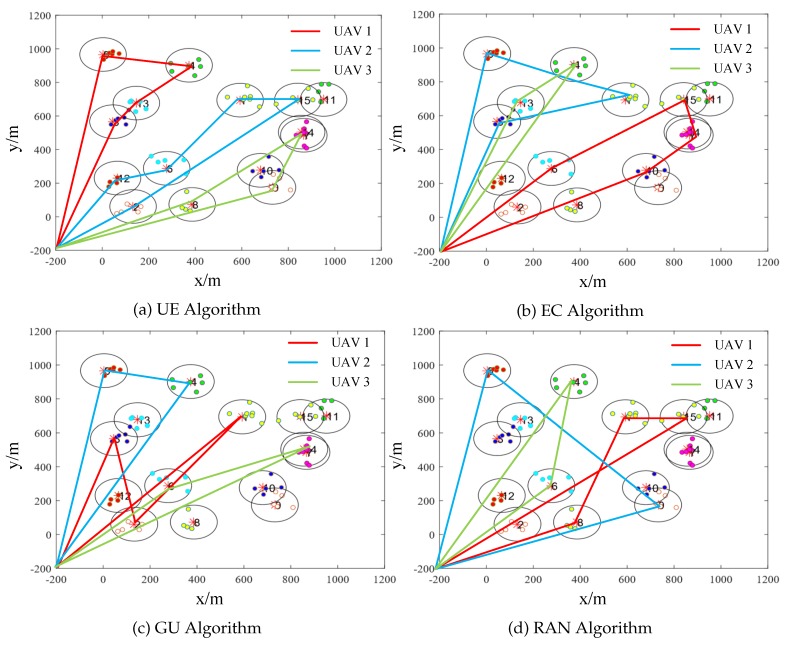
Trajectories of Three UAVs.

In this subsection, we use Figure 8 to show the resulting trajectories by each of the algorithms. The serial number of the cluster represents the importance of its coverage area. UE algorithm chooses the data collection points with the highest utility-cost ratio each time, which saves the energy of UAVs and improves the data collection utility of UAVs. Compared with the other three algorithms, our algorithm takes into account the data collection utility and the energy consumption.

## 6. Discussion

In this paper, we mainly focus on two-dimensional trajectory planning of UAVs. In fact, it is worthwhile to optimize UAV’s altitude. However, the optimization of flight altitude will bring some challenges. First, the ascend and descend of UAVs will bring extra energy consumption. Second, the flight altitude of UAVs can influence the quality of communication channel. Third, it will bring new optimization variables and increase the search space. We need to further study to solve these problems. In the future, to further improve the performance of multi-UAV data collection system, we will present a new design framework of three-dimensional UAV trajectory.

## 7. Conclusions

In this paper, we consider exploiting UAVs to collect data from sensors. The value of data collected from each sensor is different. It depends on the importance of the monitoring area of the sensor and the freshness of collected data. To improve the data collection utility, we optimize the trajectory planning, communication scheduling and sensor node association. The data collection utility is determined by the amount and value of data. First, we formulate this problem as a variant of multiple knapsack problem, which is a classical NP-hard problem. We transform the initial problem into the problem of maximizing a submodular function under energy constraints. To maximize the data collection utility, we propose a novel trajectory planning algorithm, while accounting for different value of data and different energy constraints of heterogeneous UAVs. Sufficient simulations are performed to demonstrate the validity and applicability of the proposed algorithm. The results show that the data collection utility of our algorithm can be increased by 134% at most.

## Figures and Tables

**Table 1:** Major notations.

Notation	Definition
*U*	UAV set
*S*	Sensor set
*C*	Cluster head set
*L*	UAV trajectory set
$u_{i}$	UAV *i*
$s_{j}$	Sensor *j*
$c_{a}$	Cluster head *a*
$L_{i}$	Trajectory of UAV *i*
*k*	Number of UAVs, or number of trajectories
*n*	Number of sensors
*m*	Number of cluster heads
$E_{\max,i}$	Energy budget of UAV *i*
$p_{\min,m}$	Minimum motion power
$E_{m,i}$	Motion energy consumption of UAV *i*
$p_{m,i}$	Actual motion power of UAV *i*
$b_{i}$	Length of trajectory $L_{i}$
$v_{i}$	Velocity of UAV *i*
$\eta_{i}$	Power efficiency of UAV *i*
$E_{h,i}$	Hovering energy consumption of UAV *i*
$N_{a,i}$	Amount of data collected by UAV *i* in cluster head *a*
$p_{\min,h}$	Minimum hovering power
$p_{h,i}$	Actual hovering power of UAV *i*
$R_{a,i}$	Data transmission rate between UAV *i* and cluster head *a*
$E_{c,i}$	Communication energy consumption of UAV *i*
α	Path loss exponent
$e_{x}$	Communication energy consumption parameter
$V_{j}\left(t\right)$	Value of data collected from sensor *j*
$T_{j}$	Recovery interval of sensor *j*
${t^{\prime }}_{j}$	Time of previous data collection from sensor *j*
$q_{a,i}$	Data utility of cluster head *a* collected by UAV *i*
$Q_{i}$	Data collection utility of UAV *i*
*Q*	Overall data collection utility
$v_{\max}$	Maximum velocity
$l_{i}\left(t\right)$	$u_{i}$ trajectory projected on the ground
$\overset{\cdot }{l_{i}}\left(t\right)$	Time derivative of $l_{i}\left(t\right)$
$d_{\min}$	Minimum inter-UAV distance
*H*	Altitude of UAVs

**Table 2:** Major notations.

Definition	Parameters	Values
Area size	*D*	1 km× 1 km
Minimum inter-UAV distance	$d_{\min}$	100 m
Altitude of UAVs	*H*	50 m
Maximum velocity	$v_{\max}$	20 m/s
Minimum motion power	$p_{\min,m}$	388.32 J/s
Minimum hovering power	$p_{\min,h}$	308 J/s
Power efficiency of UAV	η	70%–80%
Data transmission rate	*R*	2 Mbps
Path loss exponent	α	2
Communication energy consumption parameter	$e_{x}$	10 $pJ/(m^{\alpha }\cdot bit)$
